# Editorial

**DOI:** 10.1155/EDR.2001.1

**Published:** 2001

**Authors:** Anders A. F. Sima, Eleazar Shafrir


### EDITORIAL

As *The International Journal of Experimental Diabetes Research* enters its second year of publication, we would like to take this opportunity to thank all those who helped the journal get off to a successful start. In the first year we dedicated our attention to the publication of quality peer-reviewed research articles. We wish to thank all contributors as well as the reviewers, whose indispensable expertise helped us in the evaluation of the submitted manuscripts.

As we enter the second volume we will, of course, continue to publish primary, refereed papers, but will additionally introduce short reviews on timely topics of research. We feel that this combination will create a useful vantage point on the field that balances specialist reports with informative and insightful perspectives. Some reviews have already been invited, but we would like to encourage all those interested in contributing to contact one of us with a suggestion of title or topic. We also encourage brief communications and any correspondence regarding the published articles.

The second volume of the journal will also introduce a calendar page that will serve to highlight scientific events and meetings of interest to the Diabetes research community. Any person or organization planning a meeting, a workshop or a seminar is invited to submit the pertinent information. Submissions should include the title of the meeting, main topics, sponsoring organization, venue and address of the secretariat to whom registration should be directed. E-mail addresses and fax numbers of contributors should also be included.

We are thankful for all your support and look forward to your active participation, contributions and suggestions as we continue our effort to make this endeavor valuable to our diabetes research community.

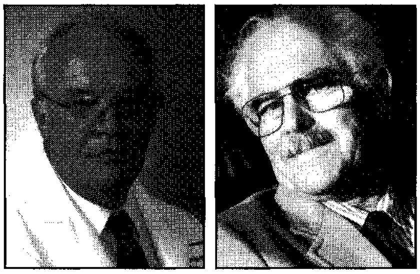

*Anders A.F. Sima, Eleazar Shafrir*
*Editors-in-Chief*

